# Supplementary material for: Optical single-channel recording of CRAC channels with HaloTag and a Ca2+-sensitive ligand
Source: bioRxiv. 2026 May 12:2026.05.08.723778. Preprint. [Version 1] doi: 10.64898/2026.05.08.723778 (PMC13192893; doi:10.64898/2026.05.08.723778)
Supplement: 1 [file NIHPP2026.05.08.723778v1-supplement-1.pdf]

bioRxiv preprint doi: https://doi.org/10.64898/2026.05.08.723778; this version posted May 12, 2026. The copyright holder for this preprint (which was not certified by peer review) is the author/funder, who has granted bioRxiv a license to display the preprint in perpetuity. It is made available under a CC-BY 4.0 International license.

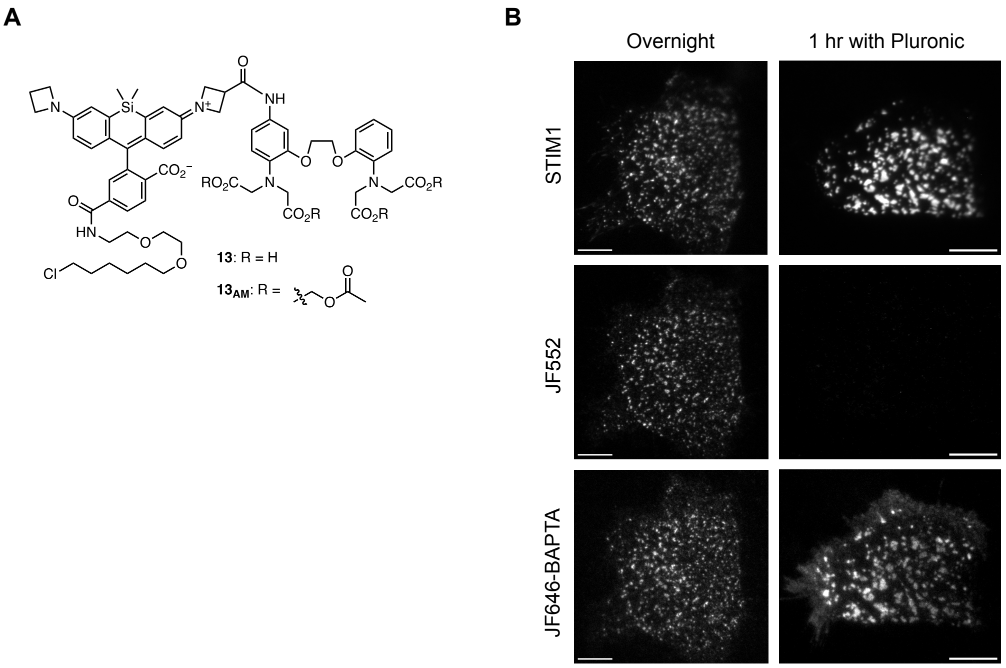

Figure S1. A method for labeling Orai1-HaloTag with the Ca^{2+}-sensitive fluor JF646-BAPTA. (A) Structure of JF646-BAPTA showing the AM esterified and de-esterified forms (Deo et al., 2019). **(B)** Testing for JF646-BAPTA labeling efficiency. HEK 293 cells were transfected with mCh-STIM1 and Orai1-HaloTag and treated with 1 µM JF646-BAPTA overnight (*left*) or for 1 h in the presence of 0.02% pluronic F-127 (*right column*), followed by exposure to 100 nM JF-552 for 10 min to label unoccupied HaloTag sites. TIRF images of the cell footprints after TG treatment show that pluronic F-127 is needed to achieve complete labeling with JF646-BAPTA. Bar=10 µm.

bioRxiv preprint doi: https://doi.org/10.64898/2026.05.08.723778; this version posted May 12, 2026. The copyright holder for this preprint (which was not certified by peer review) is the author/funder, who has granted bioRxiv a license to display the preprint in perpetuity. It is made available under a CC-BY 4.0 International license.

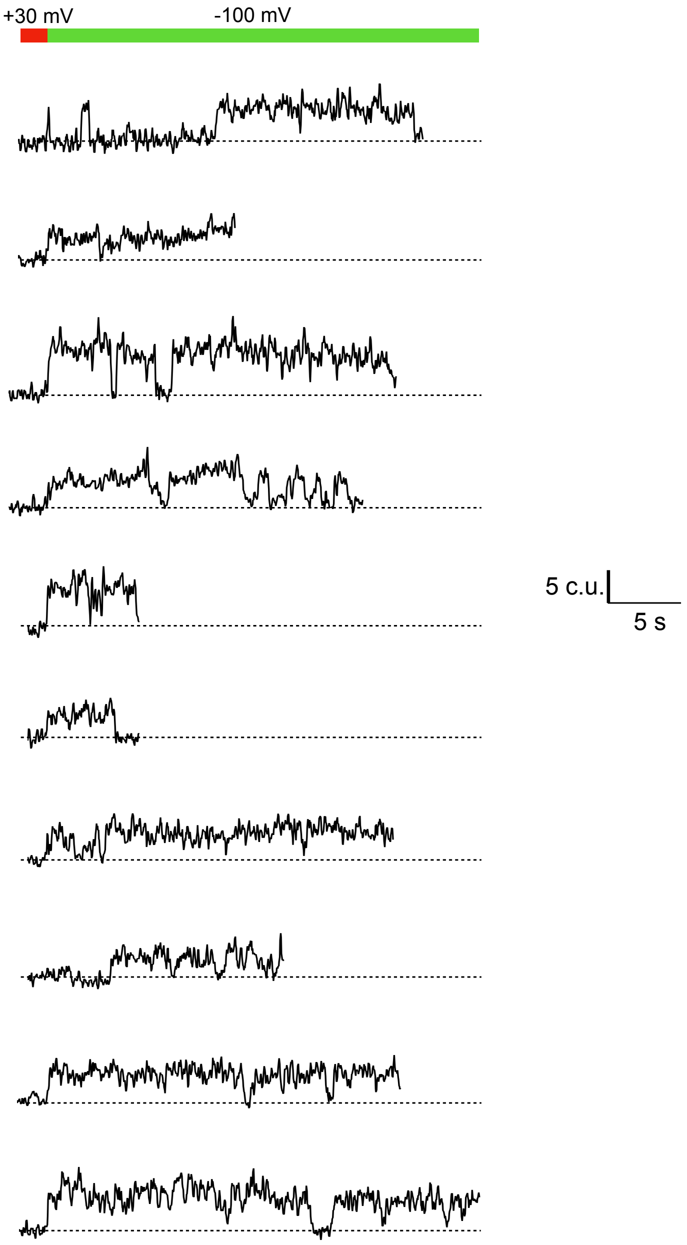

Figure S2. Single-channel optical recordings analyzed for the dwell time histograms. 36 recordings were selected for analysis based on the criteria outlined in Methods. Dashed lines indicate the intensity at +30 mV, as an indicator of the expected closed channel intensity. 33 ms sampling, 1 mW laser power, 3 frame boxcar averaging.

bioRxiv preprint doi: https://doi.org/10.64898/2026.05.08.723778; this version posted May 12, 2026. The copyright holder for this preprint (which was not certified by peer review) is the author/funder, who has granted bioRxiv a license to display the preprint in perpetuity. It is made available under a CC-BY 4.0 International license.

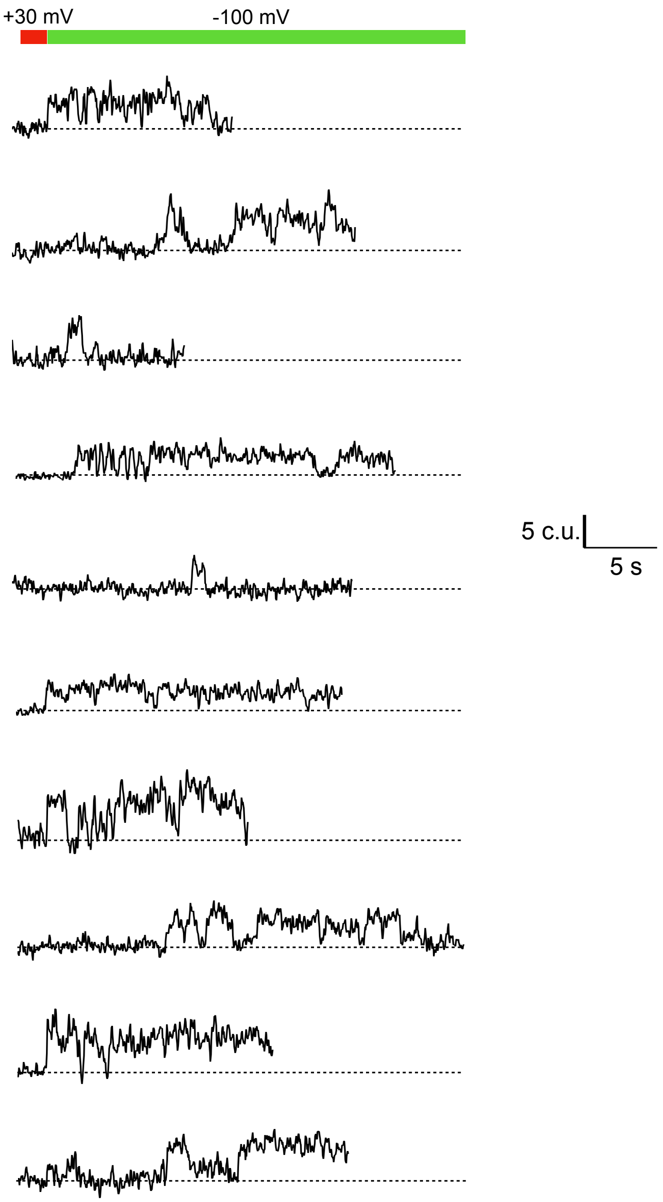

Figure S2 (cont'd). Single-channel optical recordings analyzed for the dwell time histograms. 36 recordings were selected for analysis based on the criteria outlined in Methods. Dashed lines indicate the intensity at +30 mV, as an indicator of the expected closed channel intensity. 33 ms sampling, 1 mW laser power, 3 frame boxcar averaging.

bioRxiv preprint doi: https://doi.org/10.64898/2026.05.08.723778; this version posted May 12, 2026. The copyright holder for this preprint (which was not certified by peer review) is the author/funder, who has granted bioRxiv a license to display the preprint in perpetuity. It is made available under a CC-BY 4.0 International license.

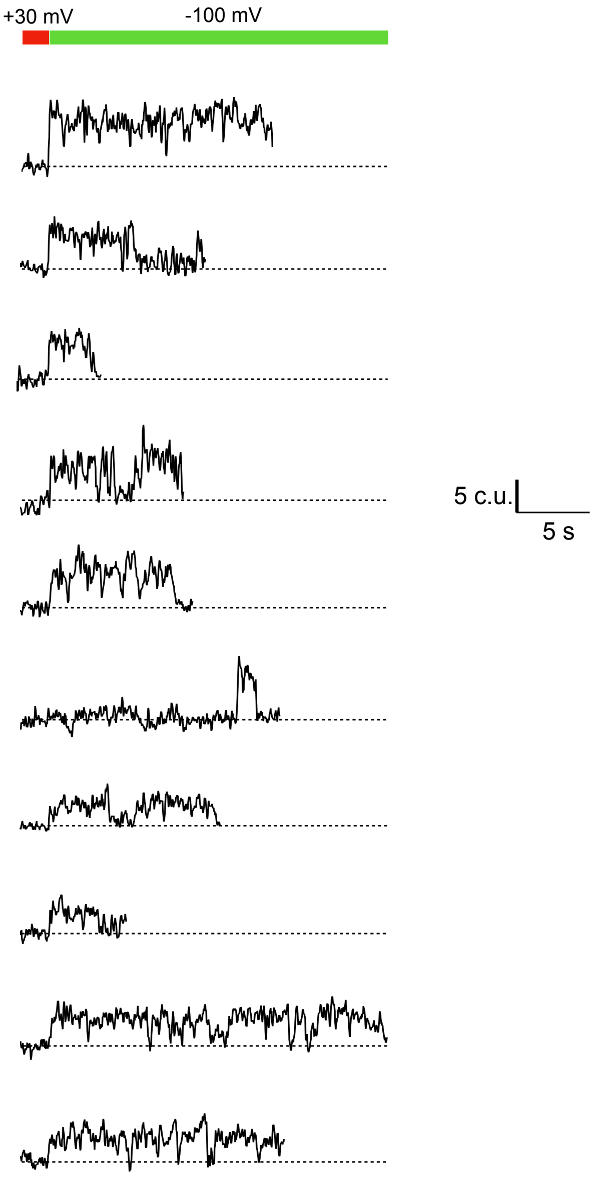

Figure S2 (cont'd). Single-channel optical recordings analyzed for the dwell time histograms. 36 recordings were selected for analysis based on the criteria outlined in Methods. Dashed lines indicate the intensity at +30 mV, as an indicator of the expected closed channel intensity. 33 ms sampling, 1 mW laser power, 3 frame boxcar averaging.

bioRxiv preprint doi: https://doi.org/10.64898/2026.05.08.723778; this version posted May 12, 2026. The copyright holder for this preprint (which was not certified by peer review) is the author/funder, who has granted bioRxiv a license to display the preprint in perpetuity. It is made available under a CC-BY 4.0 International license.

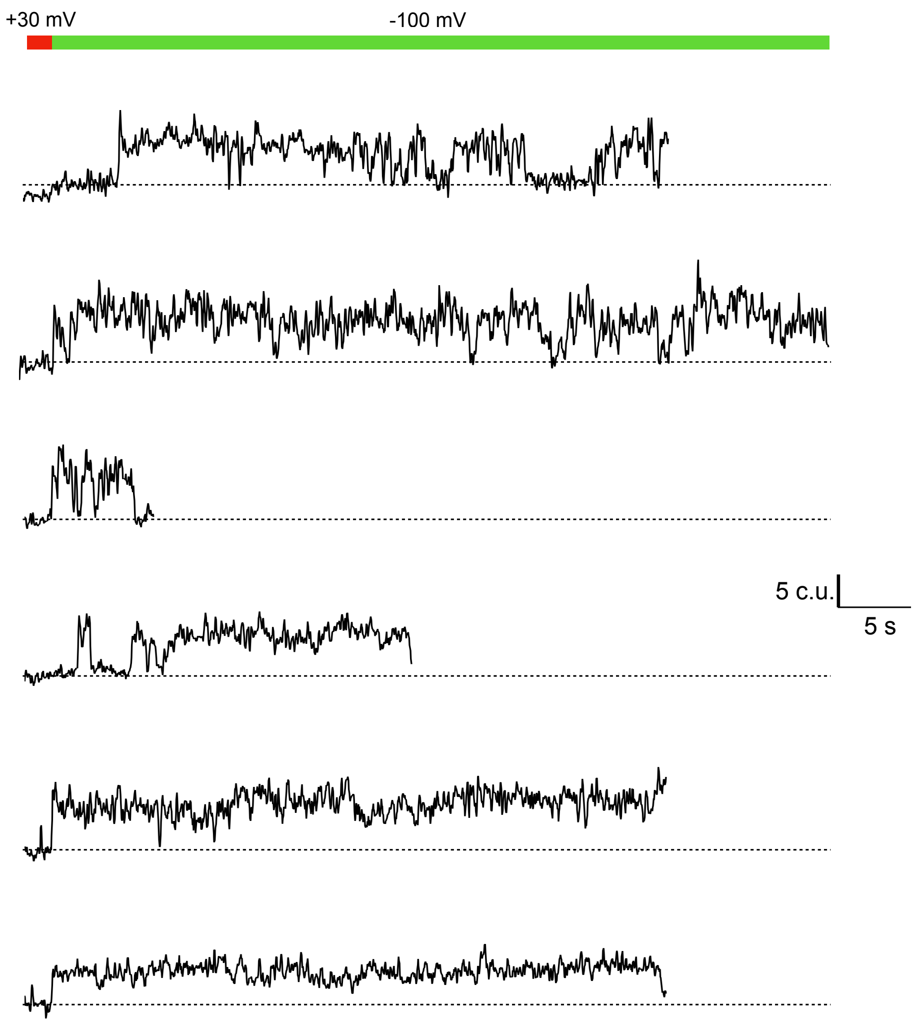

Figure S2 (cont'd). Single-channel optical recordings analyzed for the dwell time histograms. 36 recordings were selected for analysis based on the criteria outlined in Methods. Dashed lines indicate the intensity at +30 mV, as an indicator of the expected closed channel intensity. 33 ms sampling, 1 mW laser power, 3 frame boxcar averaging.
